# DNA metabarcoding and morphological macroinvertebrate metrics reveal the same changes in boreal watersheds across an environmental gradient

**DOI:** 10.1038/s41598-017-13157-x

**Published:** 2017-10-06

**Authors:** Caroline E. Emilson, Dean G. Thompson, Lisa A. Venier, Teresita M. Porter, Tom Swystun, Derek Chartrand, Scott Capell, Mehrdad Hajibabaei

**Affiliations:** 1 Natural Resources Canada, Canadian Forest Service, Great Lakes Forestry Centre, 1219 Queen St. East, Sault Ste. Marie, P6A 2E5 Canada; 2 Centre for Biodiversity Genomics @ Biodiversity Institute of Ontario & Department of Integrative Biology, University of Guelph, 50 Stone Road East, Guelph, N1G 2W1 Canada

## Abstract

Cost-effective, ecologically relevant, sensitive, and standardized indicators are requisites of biomonitoring. DNA metabarcoding of macroinvertebrate communities is a potentially transformative biomonitoring technique that can reduce cost and time constraints while providing information-rich, high resolution taxonomic data for the assessment of watershed condition. Here, we assess the utility of DNA metabarcoding to provide aquatic indicator data for evaluation of forested watershed condition across Canadian eastern boreal watersheds, subject to natural variation and low-intensity harvest management. We do this by comparing the similarity of DNA metabarcoding and morphologically derived macroinvertebrate metrics (i.e. richness, % Ephemeroptera, Plecoptera and Trichoptera, % chironomid), and the ability of DNA metabarcoding and morphological metrics to detect key gradients in stream condition linked to forested watershed features. Our results show consistency between methods, where common DNA metabarcoding and morphological macroinvertebrate metrics are positively correlated and indicate the same key gradients in stream condition (i.e. dissolved oxygen, and dissolved organic carbon, total nitrogen and conductivity) linked to watershed size and shifts in forest composition across watersheds. Our study demonstrates the potential usefulness of macroinvertebrate DNA metabarcoding to future application in broad-scale biomonitoring of watershed condition across environmental gradients.

## Introduction

Ecological indicators are deeply embedded in sustainable use, forest management policy, and third party certification systems internationally^1^, highlighting the need for practical, inexpensive, ecologically relevant and sensitive indicators in biomonitoring programs. Small freshwater streams are a ubiquitous feature of most forest regions on the Boreal Shield and represent a direct link between the terrestrial and aquatic components of forested watersheds. Physical-chemical parameters of streams have been shown to reflect disturbance history of the surrounding forested terrestrial ecosystem, for example through changes in sediment loads^2^, nutrients inputs^3^, leaf litter inputs^4^, or quantity and quality of dissolved organic matter^5^. Aquatic biota living in these streams including macroinvertebrates, fish, and algae have long been used as integrative indicators of watershed disturbance through changes in stream physical-chemical condition^6^.

Macroinvertebrates are among the most common focal groups used in biomonitoring of lotic systems and a wide array of assessment metrics that are useful in different biomonitoring scenarios have been developed based on these communities. For example, metrics based on diversity, percent of the community comprised of the taxa sensitive to disturbance (Ephemeroptera, Plecoptera, and Trichoptera; % EPT), and percent of the community comprised of resilient taxa from the family Chironomidae^7^, are often successful at detecting shifts in stream condition associated with watershed disturbance. However, Baird and Hajibabaei (2012)^8^ identified several constraints which have severely limited the utility of morphologically based macroinvertebrate metrics in broad scale biomonitoring programs over the last fifty years. These constraints include the extensive time required for field sample processing, the taxonomic expertise required to correctly identify each organism below the family level of taxonomic resolution, the potential inconsistencies amongst various observers, and the general lack of verification of morphology-based identifications^8^. Metabarcoding presents a potential solution to these morphological-based constraints, and these authors present DNA-based taxonomic identification of benthos from mixed environmental samples based on the cytochrome c oxidase subunit 1 (CO1) barcode marker using high-throughput DNA sequencing, herein referred to as DNA metabarcoding, as a potentially transformative approach to biomonitoring, biodiversity discovery, and ecosystem health assessments^9^.

The ability of DNA metabarcoding to identify known aquatic macroinvertebrate communities identified by morphological methods has been demonstrated in the literature^9–13^, and studies have highlighted the ability of DNA metabarcoding to discriminate aquatic macroinvertebrate community alpha, beta, and gamma diversity and ecological assessment metrics^14,15^. What is lacking, however, are direct validations comparing the ability of DNA metabarcoding and morphological methods to indicate gradients in specific environmental characteristics, especially gradual gradients in watershed and associated stream habitat characteristics resulting from natural and forest management induced changes. Environmental biomonitoring requires not only the identification of disturbed versus undisturbed conditions, but also the discovery of process and which environmental variables are influencing biota. In many cases disturbance is more gradual and associations between biota and the environment can help identify critical thresholds in environmental change along with multiple-variable interactions in more complex modelling scenarios. The main objective of our study is to explore the utility of DNA metabarcoding to provide aquatic indicator data for evaluation of stream health and forest integrity across eastern boreal shield watersheds subject to natural variation and the influence of low-intensity harvest management. To address our objective we: (1) Compare the similarity of DNA metabarcoding and morphological derived macroinvertebrate metrics across streams (i.e. richness, % EPT, % chironomid), (2) Identify the key gradients in stream condition that link our study streams to their forested watersheds, and (3) Compare the ability of DNA metabarcoding and morphological macroinvertebrate metrics to indicate these key gradients in stream condition.

## Results

### Similarity of DNA metabarcoding and morphological metrics

**Figure 1:**
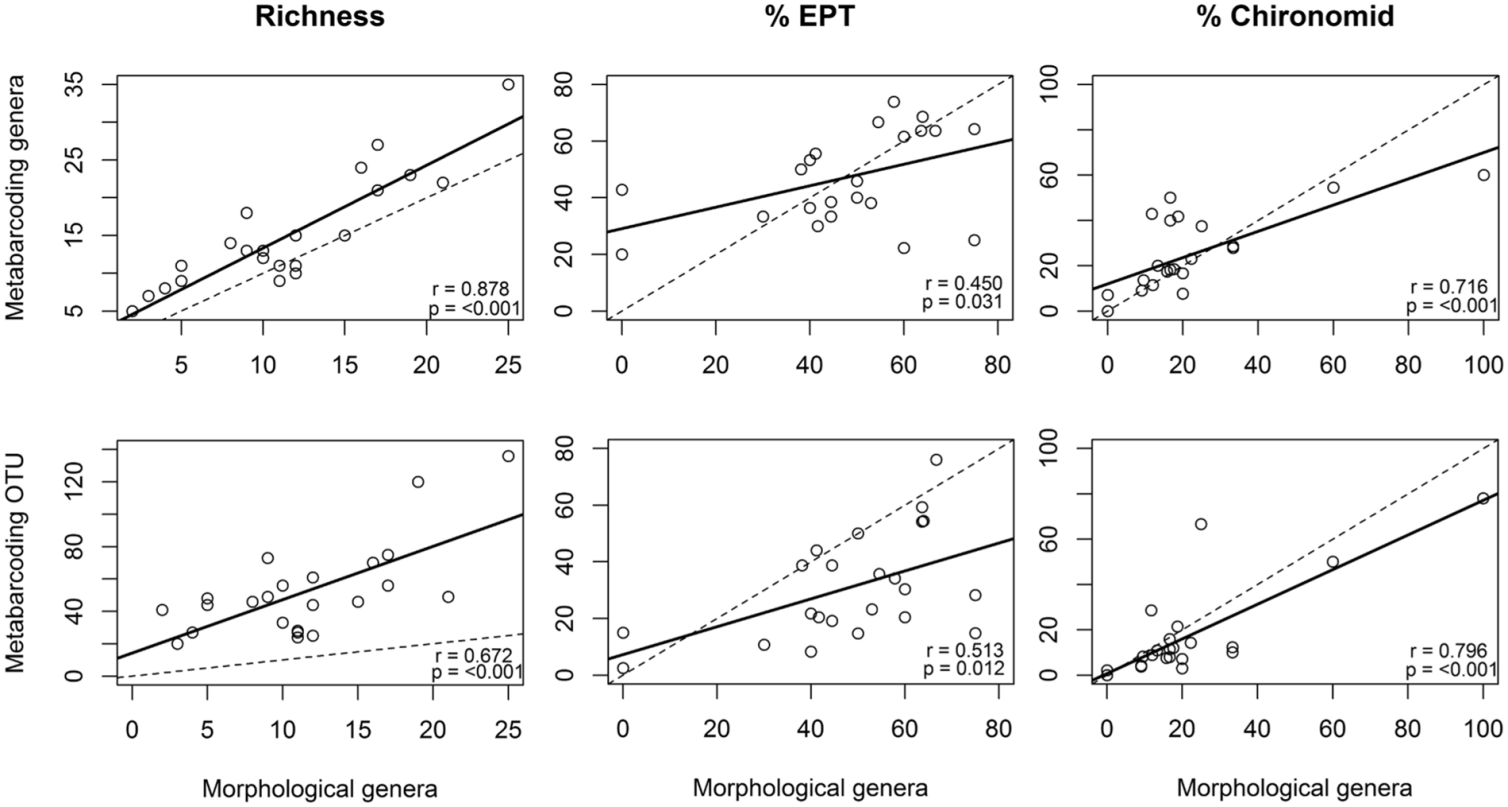
Pearson correlations between common morphological and DNA metabarcoding macroinvertebrate metrics for both genera and OTU taxonomic resolutions. The solid line represents the line of best fit, and a 1:1 line of fit is indicated (dotted line) to facilitate visual comparison between methods.

We found that common macroinvertebrate metrics (i.e. for richness, % EPT, and % chironomid) were positively correlated across our study streams for all methods (i.e. DNA metabarcoding and morphological), and taxonomic resolutions (i.e. genus and OTU) (Fig. 1). DNA metabarcoding provided higher richness values than morphological methods in most sites at the genera level, and higher richness for all sites at the OTU level as would be expected. These greater richness values, indicative of a greater number of unique taxa identified, influenced community composition metrics in two different ways, depending on the case example. In some cases, % EPT and % chironomid values decreased as the result of an increased number of non-EPT and non-chironomid taxa being detected. In other cases, DNA metabarcoding resulted in detection of comparatively more EPT and chironomid taxa, and in some cases where morphological methods detected none (see Supplementary Figs S1–S3 for site specific 1:1 taxonomic method comparisons, and Figs S4–S6 for accumulation curve method comparisons). At the genus rank, the mean DNA metabarcoding richness was 15 and 30% greater-, mean % EPT was 47.4 and 1% less-, and mean % chironomid was 24.1 and 3.2% greater-than, mean morphologically derived genera indicator metrics of 11.5, 48.4%, and 20.9%, respectively. Differences between DNA metabarcoding and morphologically derived metrics were even greater when DNA metabarcoding OTU richness was considered, with a mean richness of 52.1 being 353% greater than mean morphological genera level richness, and with mean % EPT of 31.1 and mean % chironomid of 16.7 being 17.3% and 4.2% less than means for these metrics calculated based on morphological genera level taxonomic assignments.

### Key gradients in stream condition linked to forested watershed features

**Figure 2:**
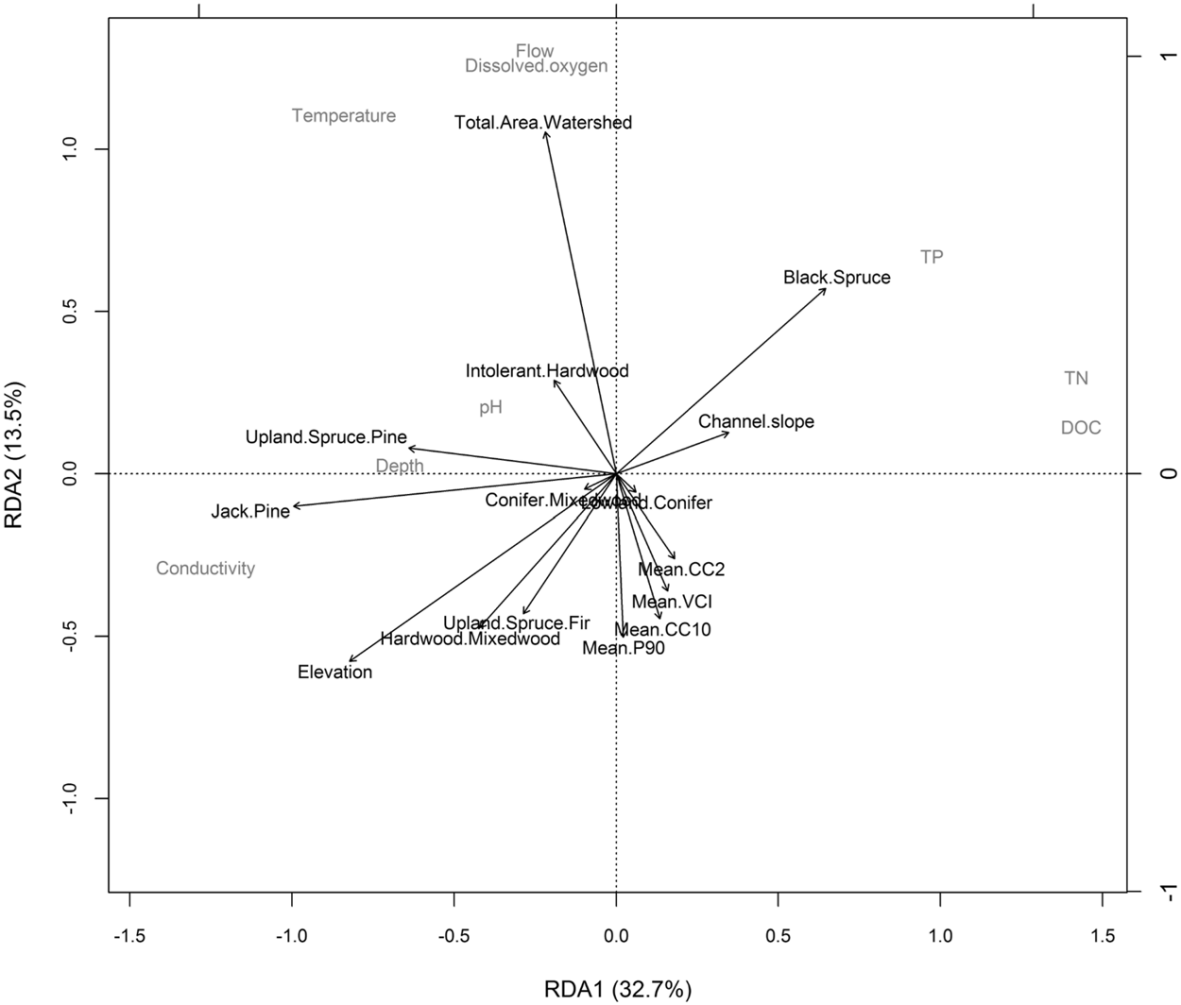
Redundancy analysis showing stream physical-chemical characteristics constrained by forested watershed features across the 23 study sites. Both axes were found to be statistically significant following a permutation ANOVA test (p = 0.001, for both RDA1 and RDA2). *DOC stands for dissolved organic carbon.

The first key gradient in stream condition linked to watershed features (RDA1; Fig. 2) represented a shift from increased stream conductivity, temperature and depth (loadings = −0.958, −0.635, and −0.505, respectively) to increased stream dissolved organic carbon, total nitrogen (TN), and total phosphorus (TP) (loadings = 1.08, 1.07, and 0.737, respectively) along a watershed gradient from higher-elevation forest with more jack pine (biplot scores = −0.773 jack pine, and −0.639 elevation) to lower elevation forest dominated by black spruce (biplot score = 0.502) (Fig. 2). The second key gradient in stream condition linked to watershed features (RDA2; Fig. 2) was represented by increased dissolved oxygen, flow, and temperature (loadings = 0.761, 0.792, 0.668 respectively) with increasing total watershed area (biplot score = 0.819) (Fig. 2). Collectively, these two watershed forest RDA axes explained 46.3% of the variation in stream physical-chemical characteristics across sites (Fig. 2).

### Utility of macroinvertebrate metrics to indicate key gradients in stream condition

**Figure 3:**
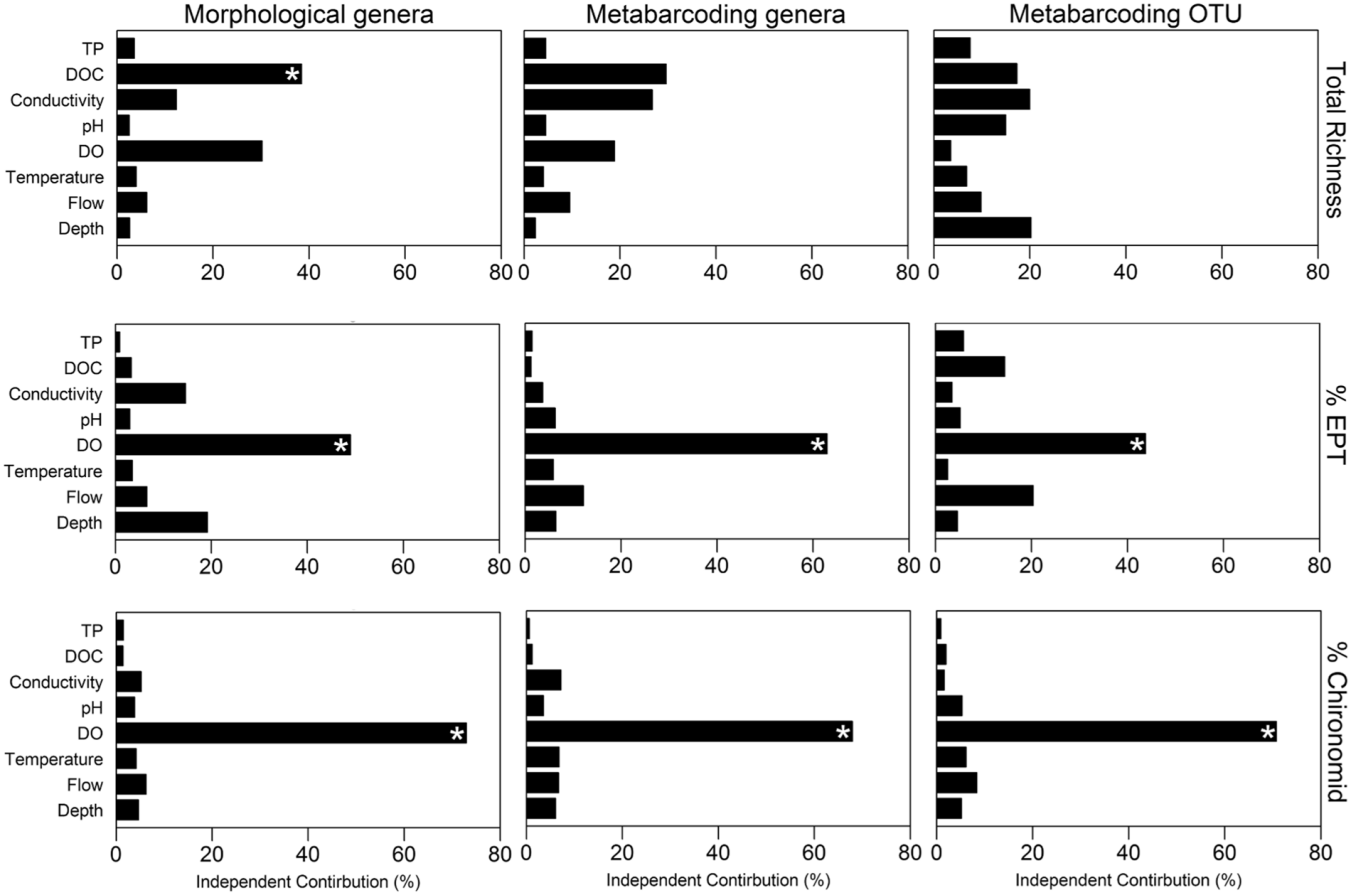
Percent independent contribution results for each macroinvertebrate metric based on hierarchical partitioning. DO stands for dissolved oxygen, DOC for dissolved organic carbon, and TP for total phosphorus. *Denotes a statistically significant (p < 0.05) independent contribution based on a negative-log-likelihood randomization test (n = 1000).

Based on hierarchical partitioning we found that dissolved oxygen and dissolved organic carbon were the stream physical-chemical variables explaining the most variation in richness, % EPT and % chironomid macroinvertebrate community composition metrics (Fig. 3). Both physical-chemical variables were also main components of the gradients in stream condition linked most strongly to forested watershed characteristics as noted above (RDA 2 and RDA1 respectively: Fig. 2). Dissolved oxygen had an independent contribution of 48.9%, 62.9%, and 43.7% for % EPT based on morphological taxonomic assignments at the level of genera, DNA metabarcoding assignments at the level of genera, and DNA metabarcoding at the OTU resolution, respectively. Similarly, variations in dissolved oxygen across streams independently contributed 72.9%, 67.9%, and 70.7% of the variance explained for % chironomid as determined by morphological genera assignments, DNA metabarcoding genera assignments, and DNA metabarcoding OTU assignments, respectively (Fig. 3). All the above listed independent contributions were found to be statistically significant (randomization test p < 0.05). Dissolved organic carbon was the only variable that had a significant independent contribution (38.4%) for morphological genera richness. DNA metabarcoding measures of richness (i.e. genera & OTU resolutions) did not have statistically significant independent contributions from any one stream physical-chemical variable, but appeared to be influenced by multivariate combinations of 4 to 5 of the 8 variables with independent contributions ranging between 10 and 30% (Fig. 3). Additionally, for all measures of richness and for % EPT composition as determined by DNA metabarcoding OTUs, joint contributions exceeded independent contributions (i.e. independent contribution/joint contribution <1) for up to 3 of the 8 explanatory variables. In contrast, joint contributions did not exceed independent contributions in any case (i.e. independent contribution/joint contribution >1 for all explanatory variables) where global models of % chironomid and % EPT were evaluated at the genera level of taxonomic resolution.

**Figure 4:**
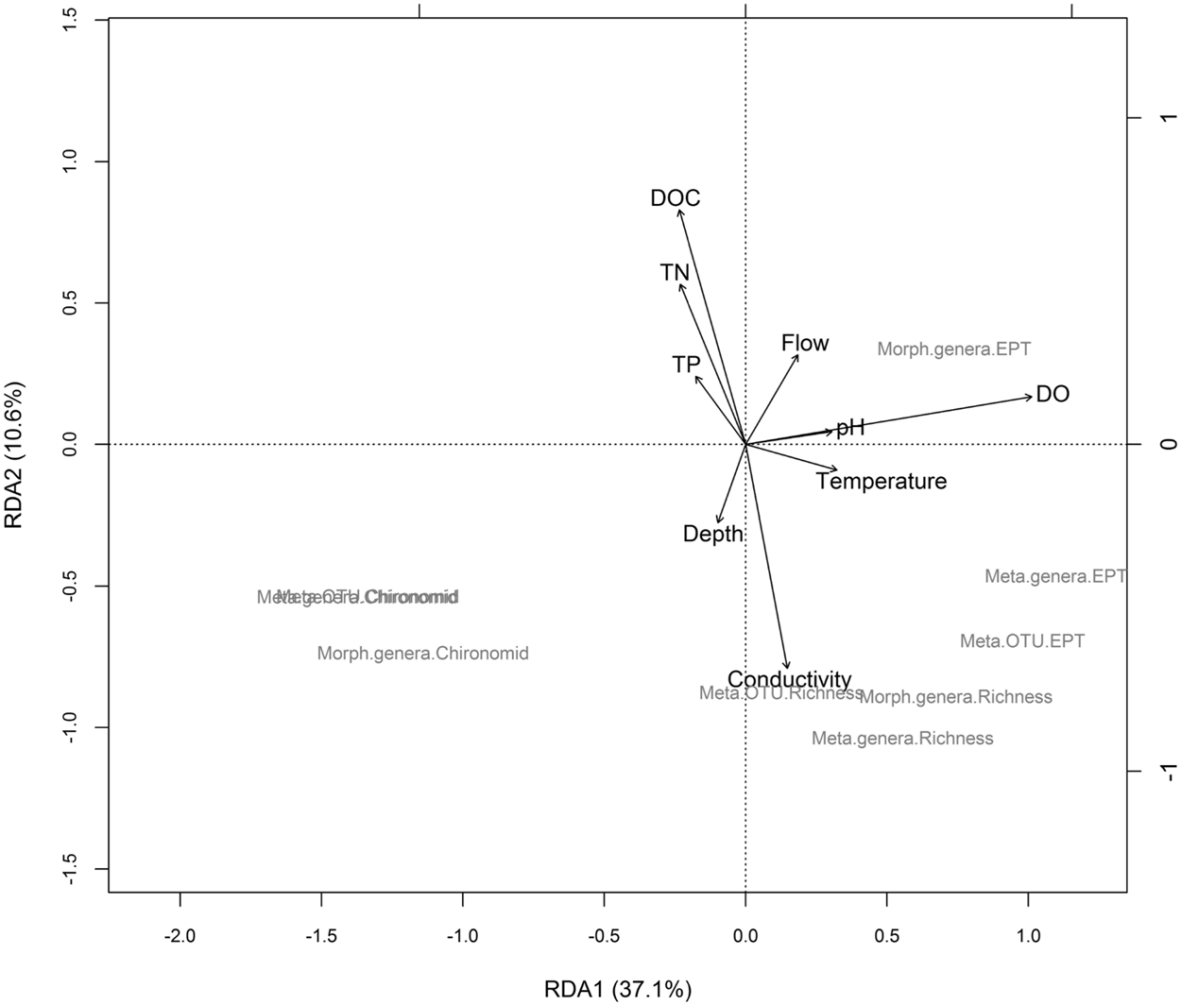
Redundancy analysis showing all macroinvertebrate metrics constrained by stream physical-chemical characteristics across the 23 study sites. Both axes were found to be statistically significant following a permutation ANOVA test (p = 0.001 and p = 0.033, for RDA1 and RDA2 respectively). Meta stands for DNA metabarcoding, Morph for morphological, DO for dissolved oxygen, and DOC for dissolved organic carbon.

Using RDA to constrain macroinvertebrate metrics by stream physical-chemical characteristics, we found that common macroinvertebrate metrics for all methods and taxonomic resolutions, were associated with the same gradients in stream condition across sites (Fig. 4). The first two statistically significant stream physical-chemical RDA axes explained a total of 47.8% of the variation in all the macroinvertebrate metrics and the main components of these gradients (i.e. dissolved organic matter, TN, conductivity and dissolved oxygen in Fig. 4) were the same as those identified as being most strongly linked to the key gradients found across the forest watersheds under study, namely jack pine and black spruce composition, watershed elevation, and watershed total area (Fig. 2).

The first gradient in stream condition (RDA1; Fig. 4), which was positively loaded with dissolved oxygen (biplot score = 0.877), explained variation in all measures of % EPT based on morphological identifications to genera, DNA metabarcoding based identifications to genera and DNA metabarcoding based identifications to OTU (loadings = 0.577, 0.858, and 0.765 respectively). Similarly, all measures of % chironomid composition in the stream macroinvertebrate communities were strongly related to dissolved oxygen including % chironomid at the level of genera based on both morphological and DNA metabarcoding data, and % chironomid based on DNA metabarcoding OTU data (loadings = −0.8690, −1.07, and −1.04, respectively). Additionally, measures of richness at the level of genera, whether determined morphologically or by DNA metabarcoding were also related to dissolved oxygen (loadings = 0.582, 0.434, respectively). The second gradient in stream condition (RDA2; Fig. 4), was positively loaded with dissolved organic carbon (biplot score = 0.719) and TN (biplot score = 0.491), and negatively loaded with conductivity (biplot score = −0.685), and explained variation in all measures of richness including morphological genera richness, DNA metabarcoding genera richness, and DNA metabarcoding OTU richness (loadings = −0.511, −0.595, and −0.499 respectively) (Fig. 4).

## Discussion

We found that common macroinvertebrate metrics showed the same relative patterns across sites regardless of method or resolution, and irrespective of the fact that DNA metabarcoding tended to identify a greater number of unique taxa than morphological methods. Furthermore, macroinvertebrate metrics from all methods and taxonomic resolutions, were associated with the strongest gradients in stream condition linked to watershed features across our study sites. These results highlight the utility of macroinvertebrate DNA metabarcoding metrics as sensitive bioindicators of environmental gradients across forested watersheds, and the potential for the CO1 BE marker to provide macroinvertebrate community data able to capture environmental change across different regions. Since the primers used to target the CO1 BE region were specifically developed to target macroinvertebrate benthos, this method could be applied to other environments where this is the target assemblage^12^. In fact, this primer set has successfully been applied in large-scale biomonitoring analysis of various sites (e.g. Gibson *et al*.^14^ used it for taxa from wetlands in Alberta). The biggest challenge for application of metabarcoding, in general, is the assumption that target taxa are already present in the CO1 reference sequence database to provide a basis for taxonomic assignment^16^. As reference databases continue to grow, we can expect the proportion of high confidence taxonomic assignments to improve. Additionally, supplementing existing reference datasets with CO1 barcode sequences obtained from locally-collected morphologically identified specimens can substantially improve taxonomic assignment success^17^.

Across all methods and taxonomic resolutions measures of overall richness were strongly associated with dissolved organic carbon, TN and conductivity, which represented the strongest gradient in stream condition linked to forested watershed features across our study sites. These changes in dissolved organic carbon, TN and conductivity across streams were associated with shifts in forest composition reflective of watershed hydrology and soil characteristics, where more lowland black spruce dominated stands were linked to higher dissolved organic carbon and nutrient concentrations, and more jack pine dominated stands linked to lower dissolved organic carbon and nutrient concentrations and greater conductivity across streams. This is consistent with soil biogeochemical differences between these forest soil types^18^, as swampy areas typical of black spruce stands are high in organic materials and thus capable of exporting greater amounts of dissolved organic carbon and nutrients to receiving streams. Likewise, well-drained mineral soils typical of jack pine stands export more inorganic ions and less dissolved organic carbon and nutrients due to low organic matter content and the increased sorption of dissolved organic carbon to mineral soil surfaces^19^. All methods and taxonomic resolutions suggest that swampy conditions typical of black spruce stands result in higher stream water dissolved organic carbon and nutrients that reduce macroinvertebrate richness, which agrees with previous research showing decreased macroinvertebrate diversity in high dissolved organic carbon and nutrient streams flooding peatlands^20^.

Despite detecting the same macroinvertebrate responses to environmental gradients, DNA metabarcoding generally found more taxa than morphological methods most likely related to the greater ability of DNA metabarcoding to identify broken, early instar, pupal and chironomidae specimens^11^. Given the ecological properties of our study system we were able to show the effectiveness of DNA metabarcoding in detecting the effects of multiple variables on macroinvertebrate richness. All measures of DNA metabarcoding and morphological richness were associated with the same multivariate axis in RDA, and had explanatory variables with joint contributions that exceeded independent contributions in hierarchical partitioning, suggesting the influence of multiple variables on overall richness measures. However, morphological genera richness identified the independent contribution of dissolved organic carbon as significant in hierarchical partitioning, where DNA metabarcoding richness measures did not find the independent contribution of DOC, or of any other individual explanatory variable to be significant. This may be attributed to the higher resolution in taxonomic information provided by genetic-based metabarcoding that is more sensitive to the detection of unique taxa and thus multiple variable gradients. Comparatively, morphological methods identify a lower number of unique taxa, and therefore may be more biased towards detecting only environmental gradients that influence macroinvertebrates collectively at lower taxonomic resolutions. Previous studies have documented the stronger discriminatory power of DNA metabarcoding macroinvertebrate communities based on the high resolution taxonomic information provided^14,21^. In our dataset, the highest resolution DNA metabarcoding OTU data detected the same environmental gradients as DNA metabarcoding genera, and did not consistently show stronger associations with these gradients. However, the increased statistical power of OTU resolution DNA metabarcoding data to detect gradients may be study specific as previous research has shown considerable variation from one region to the next in the taxonomic resolution of macroinvertebrate communities best suited to detect environmental gradients^22^. Additionally, the resolution at genus level could be influenced by the completeness of reference sequence libraries for a study site. Therefore, the high resolution OTU data that is derived from DNA metabarcoding is still an added benefit that may prove useful in teasing apart cumulative effects or multiple stressors in some cases.

All methods and taxonomic resolutions were also able to detect the same patterns in community composition in response to natural gradients in stream condition, with indicators of sensitivity and tolerance (% EPT and % chironomid) following dissolved oxygen gradients across streams. Dissolved oxygen was the second strongest gradient in stream condition linked to watershed features, and was associated with variation in watershed size and flow rate across sites. These findings are well supported in the literature, as dissolved oxygen concentrations increase with flow rates because of increased absorption of oxygen from the atmosphere from increased water movement^23^. Additionally, EPT taxa are known to be a sensitive indicator of environmental change in streams and to require high dissolved oxygen concentrations (>5 mg/L)^24^, while taxa from the family chironomidae are known to be more tolerant and thrive where EPT are in low abundance^25^. The ability of % EPT and % chironomid indicator metrics to clearly detect a key gradient in stream condition is reflective of the fact that these target taxa are known to respond to the same environmental changes (albeit in opposite directions), thereby filtering out noise that may result from including other taxa who might respond in a different or more gradual way. These findings highlight the ability of DNA metabarcoding-based methods to detect the same patterns in metrics of community tolerance and sensitivity as with morphologically derived metrics, and re-iterates the ability of DNA metabarcoding to detect the same primary responses to even subtle environmental gradients, despite finding greater richness.

Our data allowed us to demonstrate the potential use of DNA metabarcoding on a more sensitive scale because the key gradients in stream and watershed condition across our study sites lacked the discrete influence of a high-impact disturbance. Across the boreal shield and forested watersheds in general, watersheds vary in size, soil composition, topography, hydrology, and forest composition and structure. It is essential to characterize region-specific variation to define a baseline from which to base comparisons across sites and regions when assessing the effects of sustainable management practices. DNA metabarcoding of macroinvertebrate communities provides a powerful tool capable of detecting underlying gradients in forested watershed condition over space and time. Given the demonstrated ability of DNA metabarcoding to detect variation across the watersheds in this study that were subject to natural variation and low-intensity harvest management, the potential for DNA metabarcoding to detect gradients across more intensively disturbed and managed watersheds is promising.

In this study, we focused on presence-absence data to make a direct comparison between methods. However, future improvements in sequencing methods such as the optimization of mixed PCR template reactions to reduce PCR bias^26^, or capture-based sequencing that remove the need for PCR entirely^27^ may add further value to DNA metabarcoding data by allowing for abundance information to be used with greater confidence. Previous studies comparing the use of morphologically derived relative abundance with presence-absence macroinvertebrate data have found that in some cases relative abundance has comparatively stronger associations with environmental gradients^22^, while in other cases presence-absence data has been found to perform equally as well as relative abundance, for example in discriminating pollution level thresholds^28^. Other important considerations for the use of DNA metabarcoding data on large-scales, include the standardization of sample processing, DNA extraction and bioinformatic methods^29–31^. The potential development and use of DNA metabarcoding based multimetrics that integrate many different sensitivity and diversity metrics into one, as seen with stream and river macroinvertebrates across a basin in China^32^, may also prove useful.

## Conclusions

In conclusion, our results demonstrate that macroinvertebrate DNA metabarcoding data can capture the same changes in community composition, and detect the same key gradients in stream and watershed condition across sites as morphological data, ultimately leading to the same conclusions about the ecological integrity of forested watersheds. Our study also highlights the advantages of DNA metabarcoding data to not only provide common indicator response variables targeting sensitive and tolerant indicator group metrics, such as % EPT and % chironomid, but to also simultaneously provide high resolution, information rich data that may prove useful in detecting cumulative effects of environmental variables, and potentially multiple stressor scenarios. Previous literature has also documented the cost and time effective, and verifiable and reproducible nature of DNA metabarcoding data^8^, further highlighting the potential future application of macroinvertebrate DNA metabarcoding data in broad-scale biomonitoring of the ecological integrity of stream and associated forest ecosystems in Canada and internationally. Ongoing improvements and the standardization of sequencing and bioinformatic methods will further add to the value of DNA metabarcoding in biomonitoring programs.

## Methods

### Study Sites

**Figure 5:**
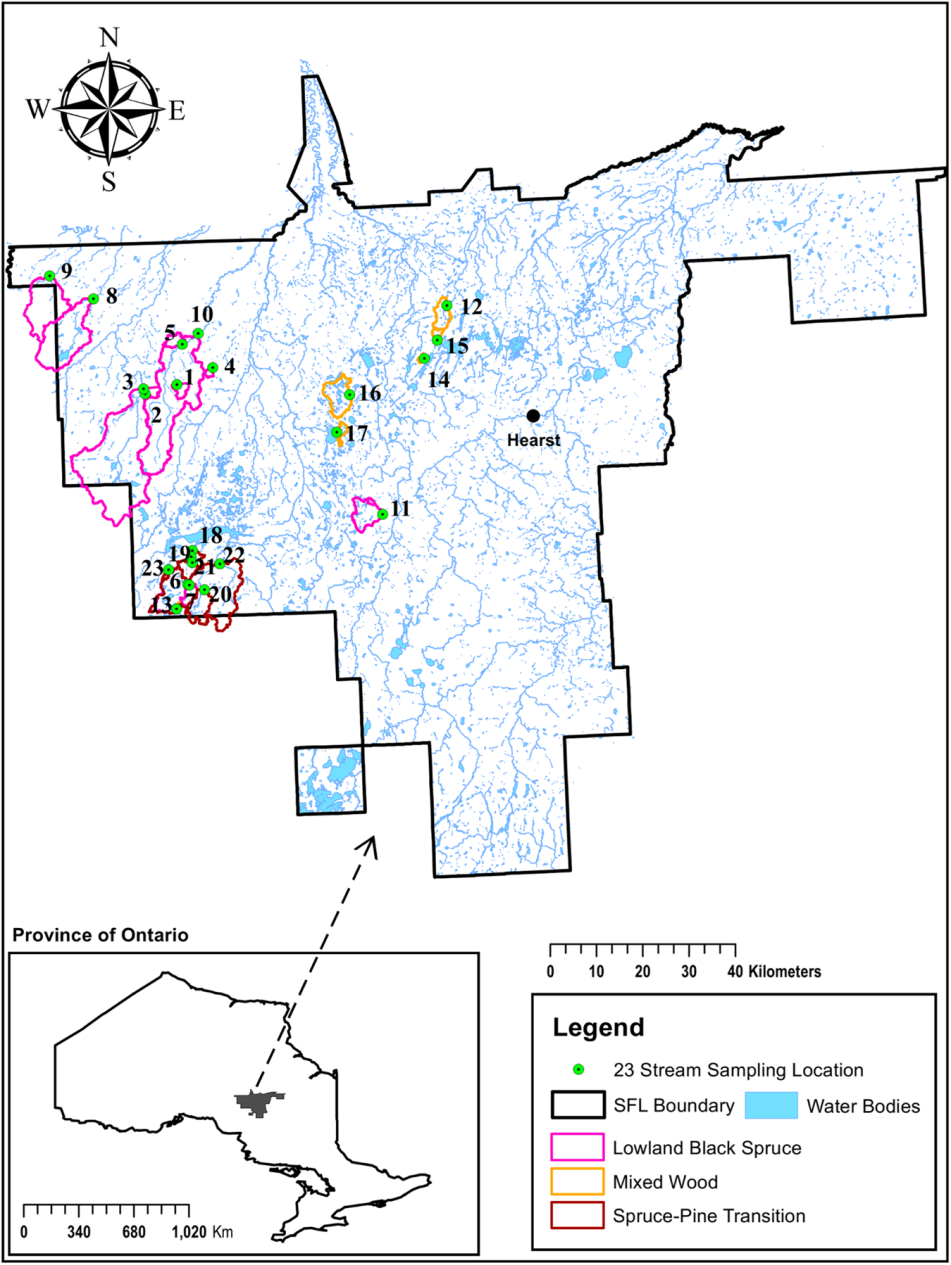
The Hearst Sustainable Forest License, showing the delineated boundaries of the 23 watersheds involved in this study, stream layers and the location of stream sampling points. The map was created in ArcMap version 10.4.1 using a base map from Land Information Ontario (https://www.ontario.ca/page/land-information-ontario), a shapefile provided by Hearst Forest Management Inc. to delineate the Hearst Sustainable Forest License area, and the GPS coordinates of the study streams.

The 23 study sites are located within the 12 thousand km^2^ Hearst Sustainable Forest License, located in northeastern Ontario (700060 E, 5490897 N 16U) (Fig. 5). The study sites contain varying proportions of black spruce (*Picea mariana* Mill. B.S.P.), cedar (*Thuja occidentalis* L.), tamarack (*Larix laricina* (Du Roi) K. Koch), white spruce (*Picea glauca* (Moench) A. Voss), jack pine (*Pinus banksiana* Lamb.), balsam fir (*Abies balsamea* (L.) Mill), and trembling aspen (*Populus tremuloides* Michx.) as described by Penner *et al*. 2013^18^, and have varying histories of low-intensity, patchy harvest and natural regeneration typical of extensive management strategies.

### Watershed characteristics

Watershed characteristics included forest composition and structural data, along with landscape features as outlined in Table 1 and were derived using enhanced forest inventory information^18^. Briefly, compositional data were derived based on interpretation of high resolution (i.e. 40 cm) digital stereo imagery, and structural characteristics and landscape features were derived from airborne light detecting and ranging (LiDAR). LiDAR capture (0.82 point/cm^2^; <30 cm vertical accuracy) was completed in 2007 and descriptor variables generated for 400 m^2^ cells allowing spatially explicit descriptions of forest structure at fine scale. Streams and drainage areas of the watersheds were delineated based on the 5 m Digital Terrain-Model derived from LiDAR data^18^.

**Table 1:** Summary of environmental characteristics across the 23 study sites including mean, minimum, maximum, and coefficient of variation (CV).

Environmental characteristic	Mean	CV (%)	Min	Max	VIF
Watershed area (km^2^)	47.1	162	0.842	278	—
Watershed elevation (m)	285	15.0	196	347	—
Watershed mean P90	8.01	29.6	3.74	13.4	—
Watershed mean VCI	0.598	14.2	0.450	0.768	—
Watershed mean CC2	66.0	20.4	45.5	91.9	—
Watershed mean CC10	21.9	54.7	1.11	49.2	—
Black spruce (%)	43.1	58.8	0.00	82.9	—
Jack pine (%)	3.25	148	0.00	14.6	—
Mixedwood conifer (%)	8.06	101	0.00	31.8	—
Mixedwood hardwood (%)	12.6	111	0.00	48.3	—
Upland spruce fir (%)	7.55	105	0.00	32.7	—
Upland spruce pine (%)	8.50	99.4	0.00	36.9	—
Lowland conifer (%)	6.14	97.4	0.00	20.1	—
Intolerant hardwood (%)	3.75	133	0.00	14.7	—
Channel slope	0.0151	100	0.00322	0.0755	—
Stream depth (cm)	41.0	75.3	4.88	150	2.02
Stream flow (m s^−1^)	0.083	15.9	0.055	0.098	1.84
Stream temperature (°C)	19.8	13.5	15.5	23.6	1.69
Stream dissolved oxygen (mg L^−1^)	5.06	33.3	1.83	7.40	2.38
Stream pH	7.85	10.5	4.29	8.47	1.52
Stream conductivity (µmho cm^−1^)	211	31.1	111	343	2.62
Stream dissolved organic matter (mg L^−1^)	20.9	45.2	1.61	36.9	2.98
Stream total phosphorus (mg L^−1^)	0.009	47.1	0.002	0.019	1.68
Stream total nitrogen (mg L^−1^)	0.612	101	0.160	0.980	—

Variance inflation factors (VIF) are included for stream physical-chemical characteristics included in hierarchical partitioning. P90 is the 90^th^ percentile of stand height, VCI stands for the vertical complexity index and represents a more even stand height as you move towards 1, and CC2 and CC10 represent percent crown closure at 2 and 10 m respectively.

### Stream physical-chemical characteristics

We collected detailed stream physical-chemical characteristics for the sampled reach of each of the study streams (Table 1). Data collected included water chemistry as determined by single mid-water column grab sample (dissolved organic carbon, TN, and TP) analyzed following standard protocol at the Canadian Forest Service water chemistry laboratory, Sault Sainte Marie^33^. Water temperature, conductivity, and dissolved oxygen were collected using a YSI model 85 hand held device (YSI Incorporated, Yellow Springs, Ohio, USA), pH collected using a HI 98127 handheld pH meter (Hanna Instruments, Woonsocket, Rhode Island, U.S.A.), and flow measured using an Single Axis EM Flow Meter (Model 801, Valeport Ltd., Totnes, Devon, UK), at a midstream location. Additionally, for each stream, depth was measured four times along each of four transects where the sample collection took place. All stream physical-chemical characteristics were collected once, at the time of macroinvertebrate sampling in 2013.

### Macroinvertebrate communities

#### Sample collection

Macroinvertebrate samples were collected during the last week of August 2013 following a modification of the standard Canadian Aquatic Biomonitoring Network protocol for wadeable streams^34^. Briefly, the samples were collected using a kick net (400 µm) over a 20 m length of stream located at least 25 m upstream of a water crossing or culvert for a standardized time of 3 minutes. The following modifications were made to prevent DNA cross-contamination among the samples: All gear including kick nets and collection containers were pre-washed with soapy water, rinsed with distilled water and ELIMINase solution, followed by ethanol, and finally exposed to UV light for at least 24 hours. Prior to sample handling, the field crew wore clean nitrile gloves, and sample sorting gear was rinsed with ELIMINase, followed by clear stream water and ethanol. After collection, the contents of the net were transferred to a bin where large inorganic and organic materials (i.e. large pieces of gravel, cobble and sticks) were discarded after first being checked for attached macroinvertebrates. The attached macroinvertebrates as well as all remaining contents of the bin were then transferred to a 250 mL bottle, covered completely with 95% ethanol, and stored in a cool, dark place at approximately 20 °C until the field sample was processed.

#### Field sample processing and morphological identification

In the laboratory, field samples were poured into decontaminated enamel trays. Under a lighted magnifying glass and using tweezers macroinvertebrates were picked from the stream matrix material and placed into clean vials. Subsequently, where possible, all macroinvertebrates from the class Insecta were identified down to genus under the microscope by Natural Resources Canada taxonomists. In total 28.8% of the specimens identified as Insecta could not be assigned to a genera based on morphology. Specimens that could not be identified down to genus included early instar or pupal specimens, damaged or broken specimens, or specimens from the family chironomidae, as the identification of chironomidae requires a comprehensive analysis by a chironomidae specialist, which is beyond the scope of routine biomonitoring analysis. Specimens from the family chironomidae were instead classified as being from the subfamily Tanypodinae, the tribe Tanytarsini, or unclassified. Unclassified chironomidae comprised 42.9% of the specimens unclassified at the genus level. To avoid DNA contamination nitrile gloves were changed, and enamel trays and all sample handling instruments were cleaned with distilled water and ELIMINase solution, rinsed with ethanol, and dried under UV light, prior to use on a different sample. The picked, morphologically identified samples were subsequently submitted for DNA metabarcoding analysis.

#### DNA Metabarcoding analysis

Each macroinvertebrate sample was homogenized in a sterile blender one sample at a time. The homogenized sample was transferred to a 50 mL falcon tube and incubated at 56 °C to evaporate excess ethanol. The homogenate was subsampled into a lysing matrix tube, and further homogenized using a MP FastPrep®-24 Instrument (MP Biomedicals Inc.; Santa Ana, California, USA) at speed 6.0 for 40 seconds. Three times the reagents suggested in the Nucleospin Tissue extraction protocol were used during the chemical lysing step, which were subdivided into three 1 mL microcentrifuge tubes. The total DNA from the homogenate was extracted from these samples using a Nucleospin Tissue Kit (Macherey-Nagel Inc.; Duren, Germany), eluting with 30 µL of molecular grade water then pooling the three reactions within each sample for a total of 90 µL of DNA extract per macroinvertebrate sample. The CO1 BE marker from each sample was amplified through a two-step polymerase chain reaction (PCR) regime using the B forward and E reverse^35^ with a standard mix of 17.5 μL molecular grade water, 2.5 μL 10x reaction buffer (200 mM Tris HCl, 500 mM KCl, pH 8.4), 1.0 μL MgCl_2_ (50 mM), 0.5 μL dNTPs (10 mM), 0.5 μL forward primer (10 mM), 0.5 μL reverse primer (10 mM), 0.5 μL Platinum *Taq* DNA polymerase (5 U/µL) (Life Technologies; Burlington, Ontario, Canada), and 2.0 μL DNA template, for a total of 25 μL per reaction. One negative control (ie. reactions with 2 μL water instead of DNA) was carried through from the first PCR to ensure PCR products were free of contamination. Reactions underwent 35 cycles of 94 °C for 40 s, 46 °C for 60 s, 72 °C for 30 s using an Eppendorf Mastercycler ep gradient S thermal cycler. PCR amplification success was visually confirmed through gel electrophoresis using a 1.5% agarose gel. Products of the first round of PCR were purified following the MinElute PCR Purification kit (Qiagen; Toronto, Ontario, Canada) standard protocol, eluting with 30 μL molecular biology grade water. Purified PCR products underwent a second round of PCR to attach the Illumina-tailed primer required for sequencing, this used the same reaction volumes and PCR conditions as the first round of PCR, with the exceptions of using Illumina-tailed primers, and 30 cycles. Illumina-tailed PCR products were then purified following the same protocol as the first round PCR products. Purified Illumina-tailed amplicons were dual indexed and sequenced on an Illumina MiSeq flow cell using the v2 sequencing chemistry (250 × 2) (Illumina; San Diego, California, USA).

In total 9 124 153 sequences were acquired and processed using a semi-automated bioinformatics pipeline described in detail in Supplementary Methods. Briefly, raw paired-end sequences (n = 8 377 424) were assembled using SeqPrep (https://github.com/jstjohn/SeqPrep), primers removed using cutadapt 1.10^36^, and sequences clustered at 98% sequence similarity into Operational Taxonomic Units (OTUs) using USEARCH^37^. Quality filtering of sequence data included minimum and maximum sequence lengths (i.e. cut offs of >300 bp, <400 bp), and removal of singletons, doubletons, sequences with 3 or more ambiguities, and chimeric sequences identified using the UPARSE-OTU pipeline. After quality filtering, a total of 1406 OTUs (n = 5 296 344 sequences) remained with an average length of 313 base pairs (See Supplementary Table S1 for summary of sequence statistics, and Fig. S7 for rarefaction curves). The Ribosomal Database Project (RDP) classifier v2.12^38^, with a custom CO1 Arthropoda training set (Porter and Hajibabaei, in prep), was chosen because of its speed and generation of confidence scores to help define good taxonomic assignments and reduce false positive identifications^16^. At the taxonomic resolution of genera a bootstrap support cut off of <50% was implemented based on previously determined leave one out testing thresholds^16^ (See Supplementary File S1 for all taxonomic assignment results). Additionally, all non-aquatic-insect OTUs were removed along with aquatic insect OTUs with bootstrap support cut offs of <20% on assignments to the class Insecta.

### Statistical approach to assess the utility of DNA metabarcoding

To compare the similarity of macroinvertebrate metrics derived from morphological and DNA metabarcoding datasets, the same metrics were independently calculated for each dataset at the genus rank for each site (n = 23), and then subsequently compared. The indicator metrics calculated included richness, and the two common community composition metrics % EPT, and % chironomid calculated based on the proportion of unique taxa classified as EPT or chironomidae divided by the total number of unique taxa for each sample. DNA Metabarcoding data at the finest level of resolution (OTU) were also used to calculate macroinvertebrate metrics to determine if higher resolution genetic data provided evidence to support similar or different conclusions. To be conservative in our comparison of methods and avoid any potential concerns with sampling inconsistencies, DNA extractions, and PCR biases^39–41^, presence-absence data was used in both DNA metabarcoding and morphological calculations of macroinvertebrate metrics. The similarity of common DNA metabarcoding and morphological macroinvertebrate metrics were then directly compared using Pearson correlation plotted with a line of best fit, and a 1:1 line of fit to visually examine the magnitude and direction of potential differences.

To identify the key gradients in stream condition that link the multivariate characteristics of our study streams to the characteristics of the forested watersheds which they drain, redundancy analysis (RDA) in the ‘vegan’ R package^42^ was used. Permutation ANOVA was run to test the significance of the RDA axes, and selected stream and watershed variables are listed in Table 1 along with summary statistics.

To compare the ability of DNA metabarcoding and morphological macroinvertebrate metrics to indicate these key gradients in stream condition across sites, associations between macroinvertebrate metrics and stream physical-chemical characteristics were examined using hierarchical partitioning in the ‘hier.part’ R package^43^, complimented with RDA. Hierarchical partitioning allows for the exploration of the relative influence of each explanatory variable on a response variable by calculating the independent and joint contributions of each individual explanatory variable in a global model, in all possible model combinations and then taking the mean^44^. RDA compliments hierarchical partitioning because it allows for associations between all DNA metabarcoding and morphological macroinvertebrate metrics and all stream physical-chemical metrics to be visualized and explored simultaneously^45^. The function ‘rand.hp’ was used to test the significance of independent contributions using a randomization test with negative log-likelihood (n = 1000), and Permutation ANOVA was run to test the significance of the RDA axes.

Prior to hierarchical partitioning, the variance inflation factors (VIF) of all explanatory variables were assessed using the ‘vif’ function in the ‘car’ R package^46^, to avoid issues of multicollinearity^47^. All key explanatory stream physical-chemical variables selected for use in hierarchical partitioning had VIF values of <3 (Table 1). TN was excluded from the hierarchical partitioning analyses because of its high VIF value (>5) indicative of its positive association with dissolved organic carbon concentrations across streams. Prior to all analyses, variables were transformed where necessary using log, square root, or logit transformations for percentages to meet normality assumptions. All statistical analyses were run in R version 3.3.2^48^.

### Data availability

Raw sequence data was deposited at the National Centre for Biotechnology Information (NCBI) Short Read Archive Project number SRP117571. Raw environmental data are also included (Supplementary File S2), along with fasta files of OTUs for each site (Supplementary File S3).

## Electronic supplementary material

Supplemental Information

File S1-S3
