# Author Correction: DNA metabarcoding and morphological macroinvertebrate metrics reveal the same changes in boreal watersheds across an environmental gradient

**DOI:** 10.1038/s41598-018-22978-3

**Published:** 2018-03-12

**Authors:** Caroline E. Emilson, Dean G. Thompson, Lisa A. Venier, Teresita M. Porter, Tom Swystun, Derek Chartrand, Scott Capell, Mehrdad Hajibabaei

**Affiliations:** 1 Natural Resources Canada, Canadian Forest Service, Great Lakes Forestry Centre, 1219 Queen St. East, Sault Ste. Marie, P6A 2E5 Canada; 2 Centre for Biodiversity Genomics @ Biodiversity Institute of Ontario & Department of Integrative Biology, University of Guelph, 50 Stone Road East, Guelph, N1G 2W1 Canada

Correction to: *Scientific Reports* 10.1038/s41598-017-13157-x, published online 06 October 2017

This Article and the accompanying Supplementary Information file contain a repeated typographical error where,

“CO1 BE”

should read:

“CO1 BR5”

Additionally, in the Methods section under the subheading ‘DNA Metabarcoding analysis’,

“The CO1 BE marker from each sample was amplified through a two-step polymerase chain reaction (PCR) regime using the B forward and E reverse^35^…”

should read:

“The CO1 BR5 marker from each sample was amplified through a two-step polymerase chain reaction (PCR) regime using the B forward and ArR5 reverse^14^…”

Finally, this Article contains an error in Supplementary Figure S1, where the order labels are misaligned with respect to the taxa. The correct Figure S1 appears below as Figure 1.

**Figure 1:**
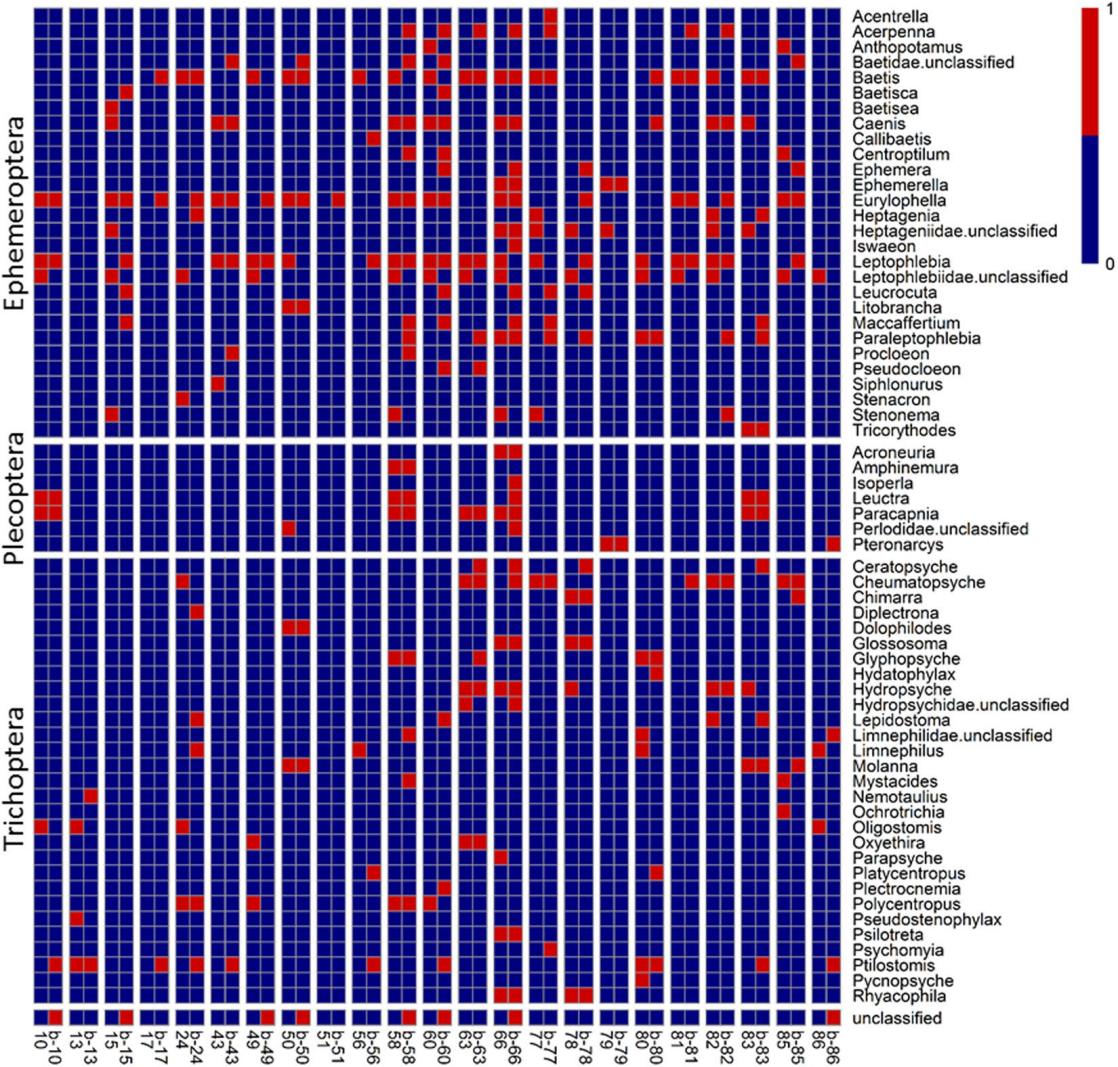
.

