# Supplementary material for: DNA metabarcoding and morphological macroinvertebrate metrics reveal the same changes in boreal watersheds across an environmental gradient
Source: Sci Rep. 2017 Oct 6;7:12777. doi: 10.1038/s41598-017-13157-x (PMC5630640; doi:10.1038/s41598-017-13157-x)
Supplement: Supplementary file 1 — Supplemental Information [file 41598_2017_13157_MOESM1_ESM.pdf]

# Supplementary Information

## DNA metabarcoding and morphological macroinvertebrate metrics reveal the same changes in boreal watersheds across an environmental gradient

Caroline E. Emilson[1*], Dean G. Thompson[1], Lisa A. Venier[1], Teresita M. Porter[1,2], Tom Swystun[1], Derek Chartrand[1], Scott Capell[1] and Mehrdad Hajibabaei[2*]

## Supplementary Methods

### *Bioinformatic pipeline and macroinvertebrate data handling*

Sequences were processed using a semi-automated bioinformatics pipeline as follows: Raw paired-end sequences were assembled using SeqPrep (https://github.com/jstjohn/SeqPrep) with default settings except that we required an overlap of 25bp and a minimum Phred quality score of at least 20. Primers were removed in a two-step process using cutadapt 1.10 [1]. First, the forward primer was removed using default settings (allowing up to a 10% mismatch between the primer and sequence).  Second, the reverse primer was removed using default settings except that we specified a minimum sequence length of 300bp (after trimming), a maximum sequence length of 400bp, a minimum Phred score of 20, and we discarded sequences with 3 or more ambiguities. FASTQ files were converted to FASTA files using mother 1.34.4 [2]. Sequences were clustered into operational taxonomic units (OTUs) using USEARCH [3]. For each sample, sequences were dereplicated (clustered with 100% sequence similarity) and singletons (clusters comprised of a single sequence) were removed. This was done to remove potentially artefactual sequences containing sequencing errors [4]. Remaining sequences were sorted by decreasing size, clustered into OTUs with 98% sequence similarity using the UPARSE-OTU pipeline that also removes putatively chimeric sequences. Doubletons (OTUs comprised of two sequences) were then removed as being potentially artefactual. Remaining OTUs were classified using the Ribosomal Database Project (RDP) classifier v2.12 [5] with a custom CO1 Arthropoda v3 training set (Porter et al., in prep). Briefly, the RDP classifier is a naïve Bayesian classifier originally developed to process 16S rRNA sequences providing bootstrap confidence estimates for each

taxonomic assignment. We trained the classifier using our own corpus of 685,651 CO1 Arthropoda sequences mined from GenBank to make taxonomic assignments to the genus rank.

Previous leave one out testing allowed us to delimit bootstrap support cutoffs that would yield correct assignments at least 99% of the time (genus=50%, family=30%, order=20%) with the assumption that the query sequence is present in the training set. Rarefaction curves were plotted to assess sequencing depth for each sample using the 'rarecurve' function in the VEGAN package in R [6]. Additional accumulation curves were plotted to assess sampling effort across all samples identified using CO1 BE metabarcoding and traditional morphology using the 'specaccum' function in the VEGAN package in [R] [6].

All non-aquatic-insect OTUs were removed, along with OTUs classified to the class Insecta with confidence values of < 20 % (total n = 59, 4.2%). Classifications with confidence values of < 20 % for order, < 30% for family, or < 50% for genus were set to unclassified. For both morphological and DNA metabarcoding classification, if the previous taxonomic level was classified with confidence this information was included (i.e. Insecta unclassified for low-confidence order, order unclassified for low-confidence family, or family unclassified for low-confidence genus classification). Presence-absence matrices were then generated from each of the global morphological and DNA metabarcoding datasets (i.e. dataset including all taxonomic information by site) using the splist2presabs function [7]. Finally, from the presence-absence matrix richness, % EPT and % Chironomid were calculated at each taxonomic resolution (including OTU) for each site.

## Supplementary Figures

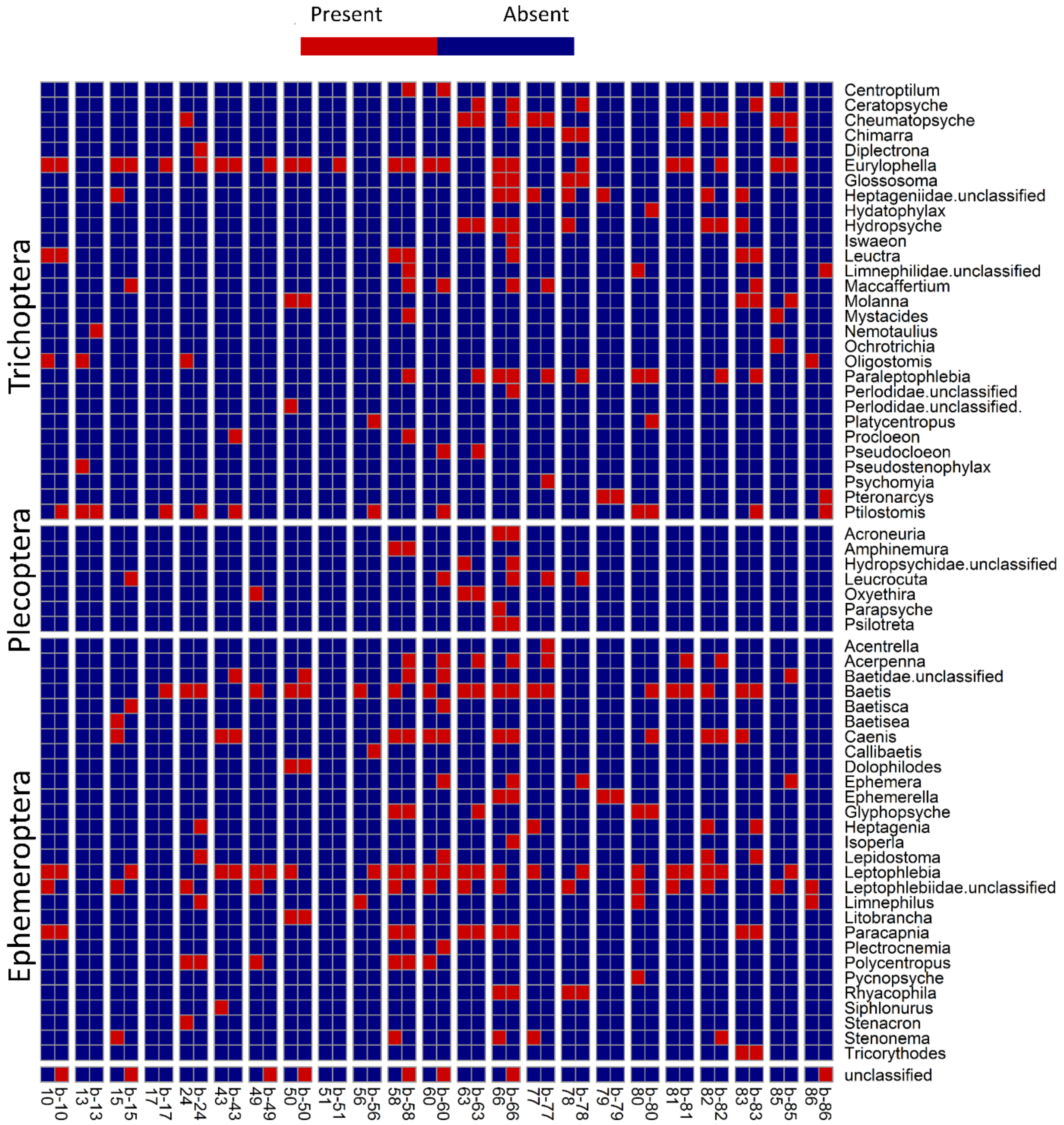


**Figure S1.** Heatmap showing the presence/absence (red/blue) across Hearst sites of all genera (rows) from the orders Ephemeroptera, Plecoptera, and Trichoptera (EPT). Morphological and DNA metabarcoding results are paired for each site (columns) with -b before the site number representing DNA metabarcoding.

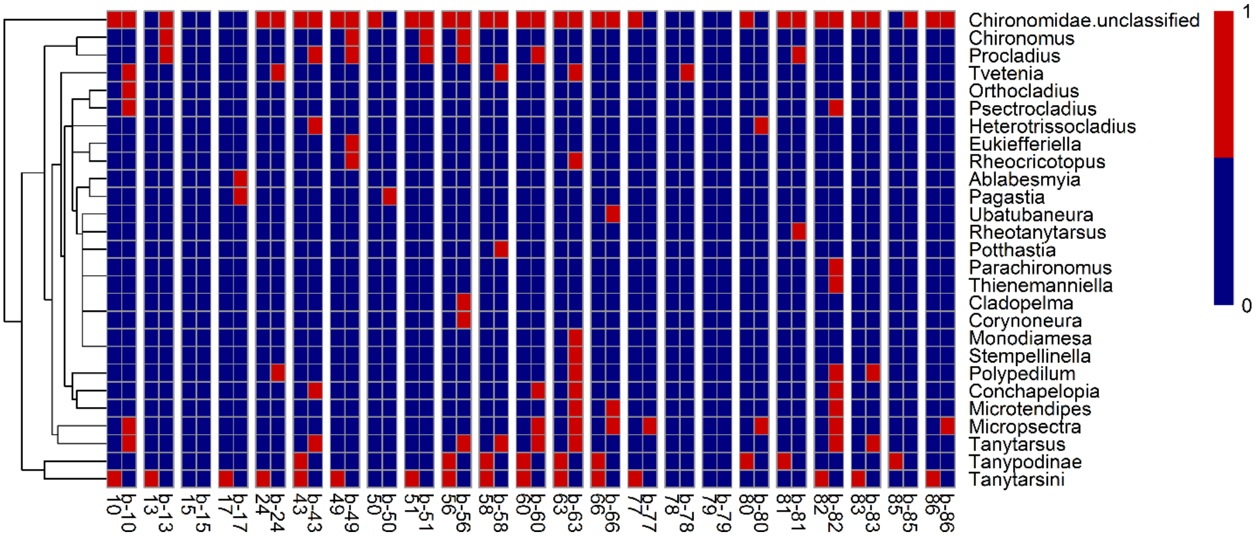


**Figure S2.** Heatmap showing the presence/absence (red/blue) across Hearst sites of all genera (rows) from the family chironomidae. Morphological and DNA metabarcoding results are paired for each site (columns) with -b before the site number representing DNA metabarcoding.

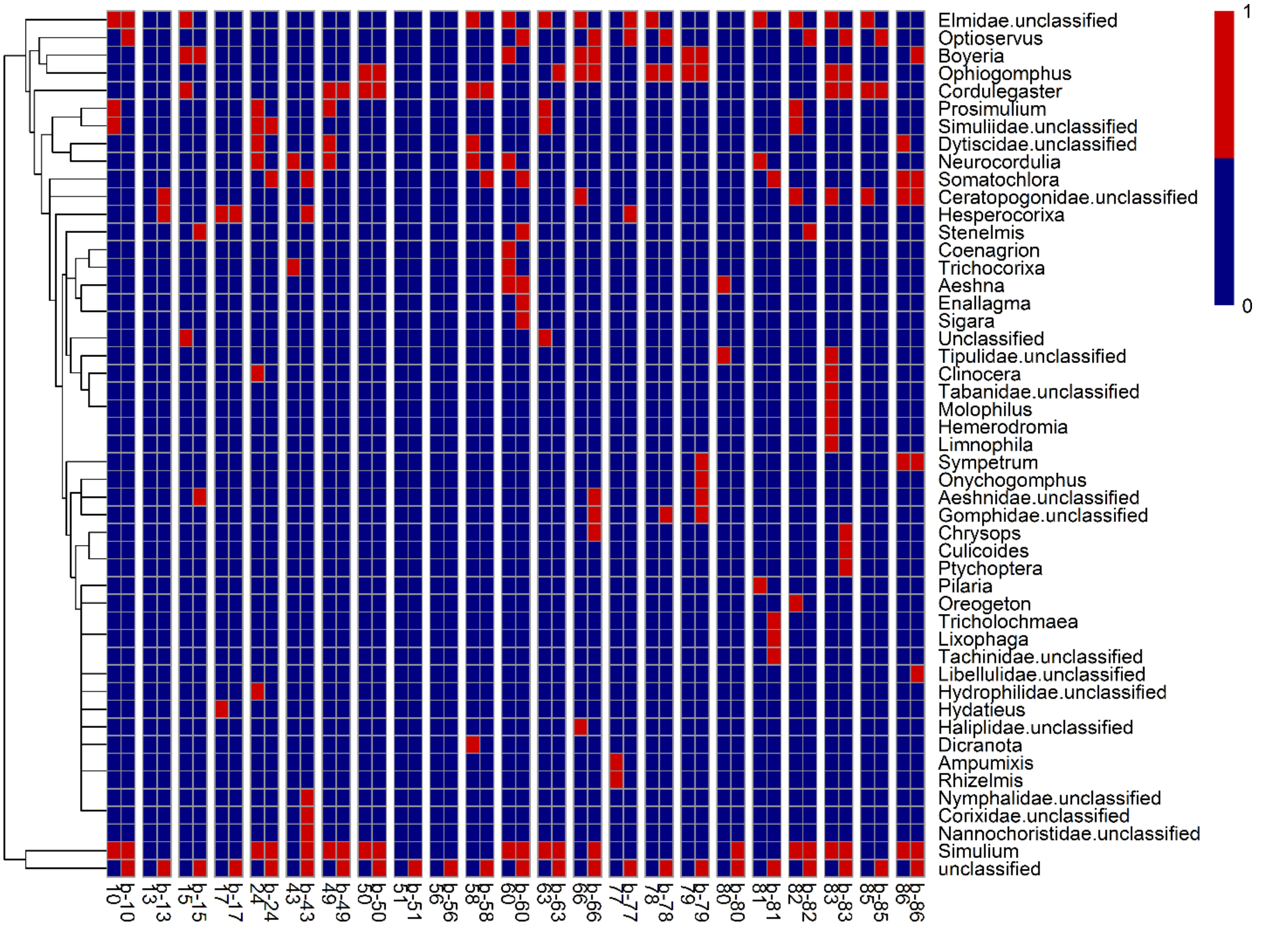


**Figure S3.** Heatmap showing the presence/absence (red/blue) across Hearst sites of all genera (rows) excluding EPT and chironomidae genera. Morphological and DNA metabarcoding results are paired for each site (columns) with -b before the site number representing DNA-metabarcoding.

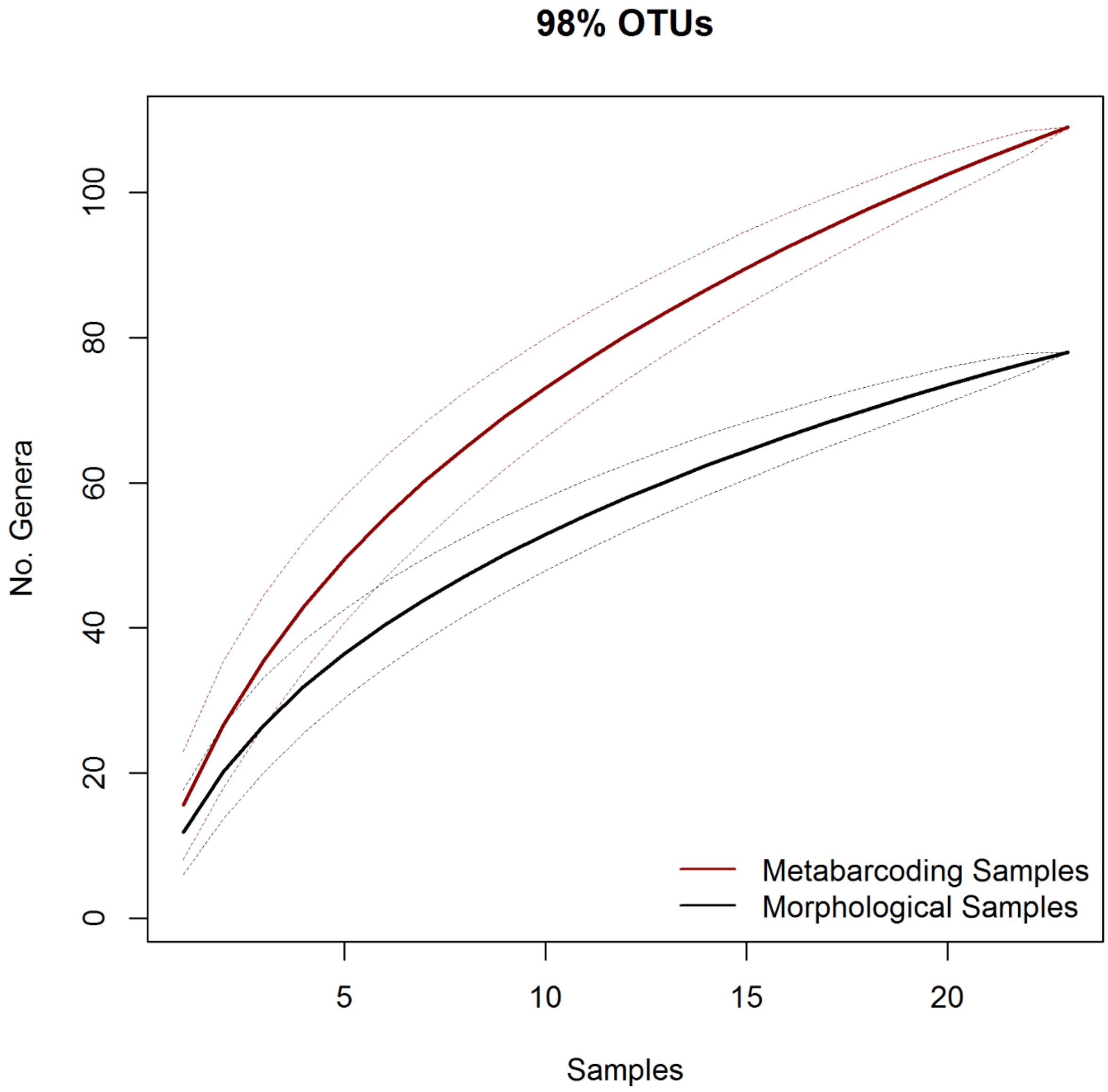


**Figure S4**. Accumulation curve of unique genera from samples identified through CO1 BE metabarcoding and traditional morphology across the 23 study sites.

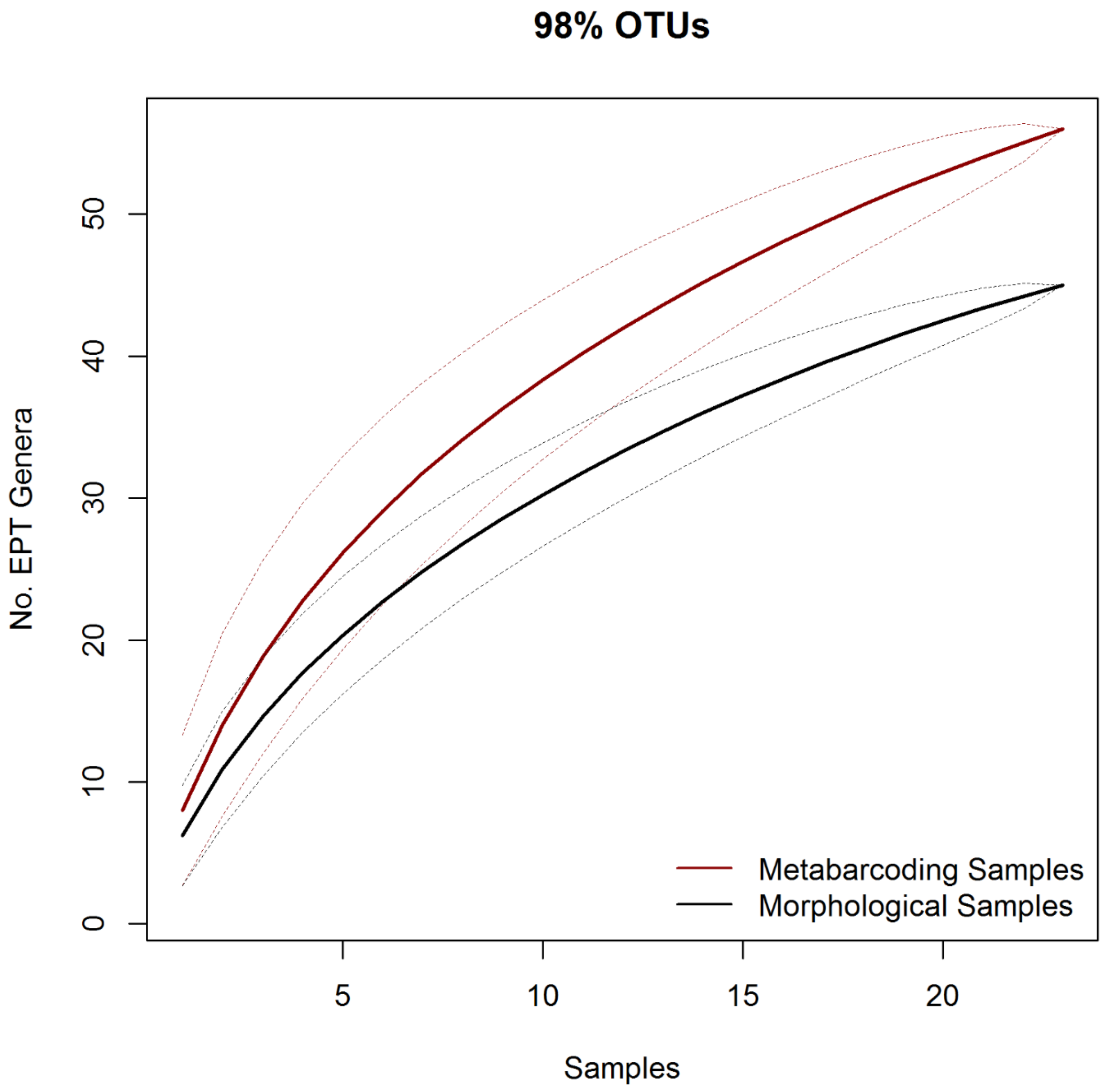


**Figure S5**. Accumulation curve of unique genera from the orders Ephemeroptera, Plecoptera, and Trichoptera (EPT) for samples identified through CO1 BE metabarcoding and traditional morphology across the 23 study sites.

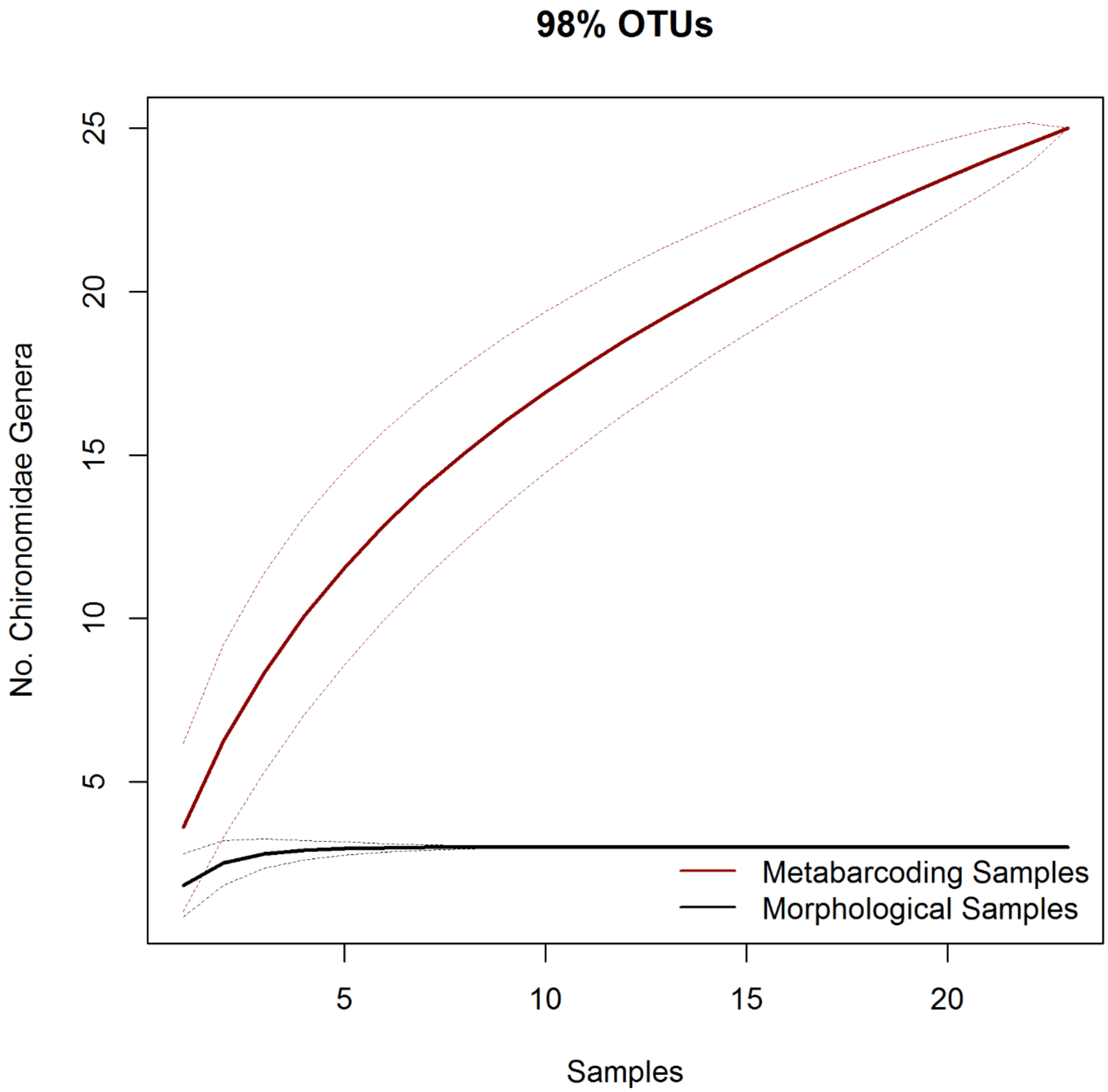


**Figure S6**. Accumulation curve of unique genera from the chironomidae family for samples identified through CO1 BE metabarcoding and traditional morphology across the 23 study sites.

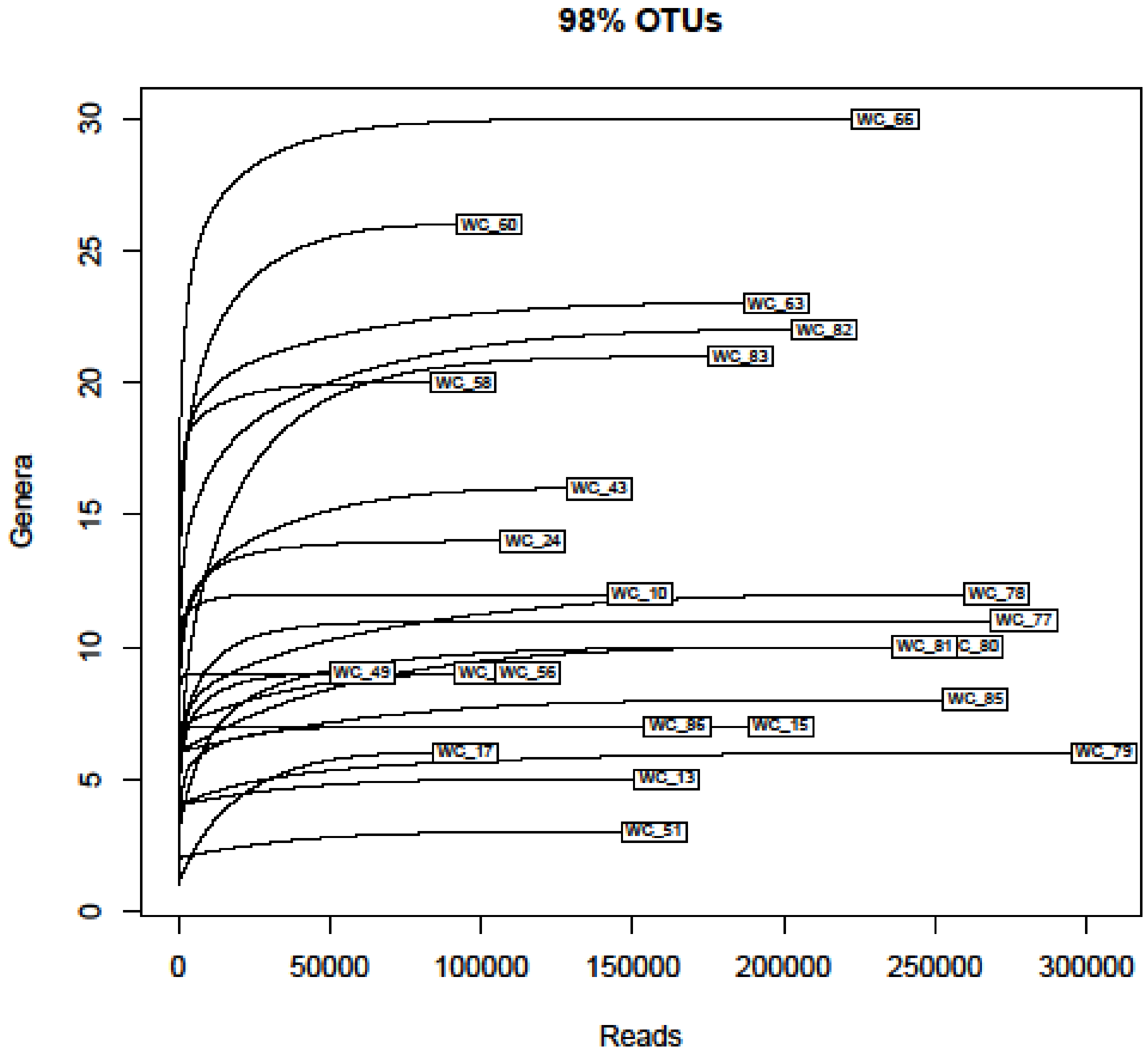


**Fi**
**gure S7.** Rarefaction curves of taxonomically assigned OTUs (98% sequence similarity cutoff) for each sample showing number of unique genera identified as the number of sequences, sequenced increases. Levelling off of the curves ensures that an adequate sequencing depth was achieved.

# Supplementary Tables

**Table S1:** Counts of reads, OTUs, and sequence length at different stages of processing for the 23 macroinvertebrate samples that were sequenced.

| | Total number **reads** | **Ave**rage number **reads per sample** | **Ave**rage **read length (bp)** | Total number **OTUs** | **Ave**. **OTU length (bp)** | **Total** number **reads in all OTUs** |
|---|---|---|---|---|---|---|
| **Raw reads*** | 9,124,153 | 396,702 | ~243 | | - | - |
| **Paired reads** | 8,377,424 | 366,214 | ~342 | | - | - |
| **Forward primer trimmed** | 8,365,903 | 363,735 | ~322 | | - | - |
| **Reverse primer trimmed** | 7,956,090 | 345,917 | ~313 | | - | - |
| **OTUs**** | - | - | - | 1,406 | ~313 | 5,296,344 |

*9,124,153 R1 and 9,124,153 R2 paired end reads

**OTUs defined by 98% sequence similarity, singletons and doubletons removed

## Supplementary References


1. Martin, M. Cutadapt removes adapter sequences from high-throughput sequencing reads. *EMBnet.journal* **17,** 10–12 (2011).
2. Schloss, P. D. *et al.* Introducing mothur: Open-source, platform-independent, community-supported software for describing and comparing microbial communities. *Appl. Environ. Microbiol.* **75,** 7537–7541 (2009).
3. Edgar, R. C. Search and clustering orders of magnitude faster than BLAST. *Bioinformatics* **26,** 2460–1 (2010).
4. Reeder, J. & Knight, R. The 'rare biosphere': a reality check. *Nat. Methods* **6,** 636–637 (2009).
5. Wang, Q., Garrity, G. M., Tiedje, J. M. & Cole, J. R. Naive Bayesian classifier for rapid assignment of rRNA sequences into the new bacterial taxonomy. *Appl. Environ. Microbiol.* **73,** 5261–5267 (2007).
6. Dixon & Palmer. VEGAN, a package of R functions for community ecology. *J. Veg. Sci.* **14,** 927–930 (2003).
7. Barbosa, A. M. fuzzySim: Fuzzy similarity in species' distributions. R package, version 0.1. (2014).
